# Virtual reality versus theoretical training in CPR among adolescents: a randomized trial with a one-year longitudinal follow-up

**DOI:** 10.1016/j.resplu.2025.101178

**Published:** 2025-11-25

**Authors:** Ana Belén Ocampo Cervantes, Carmen Amalia López López, Cristina Cerezo Espinosa, Robert Greif, Manuel Pardo Rios, Daniel Guillén Martínez

**Affiliations:** a Grupo de Investigación de Nuevas Tecnologías para la Salud, UCAM Universidad Católica de Murcia, Región de Murcia, Spain; b Hospital Universitario Santa Lucia, Cartagena, Murcia, Spain; c Gerencia de Urgencias y Emergencias Sanitarias 061 de la Región de Murcia, Spain; d Faculty of Medicine, University of Bern, Bern, Switzerland

**Keywords:** Cardiopulmonary resuscitation, Virtual reality, Secondary education, Self-efficacy, Attitude, Immersive learning

## Abstract

**Objective:**

This study evaluated the effectiveness of a virtual reality (VR)-based cardiopulmonary resuscitation (CPR) training program compared with traditional theoretical instruction and a non-intervention control group, on improving and retaining knowledge, attitudes, and self-efficacy of secondary school students. Design

Randomized trial with three parallel groups and longitudinal follow-up at baseline (T0), immediate post-intervention (T-Immediate), one-month follow-up (T-Month), and twelve-month follow-up (T-Year). Participants: 102 secondary school students (aged 14–15 years) from the Region of Murcia, Spain were randomly assigned to three groups (*n* = 34 each). Intervention: Group 1: immersive VR training (interactive 360° video); Group 2: Traditional theoretical training; Group 3: control (no intervention). The primary outcome was the objective CPR knowledge. The secondary outcomes were attitude towards CPR, perceived self-efficacy, and system usability (only in the VR group), and correlations between motivational variables and knowledge.

**Results:**

All groups showed changes after the intervention, but improvements were significantly greater in the two instructional groups. The VR group achieved the highest scores in knowledge, attitude, and self-efficacy immediately after the intervention (*p* < 0.001), and maintained more stable results at 1-month and 1-year follow-ups as compared to the theoretical group, whose initial gains declined over time. The control group did not show relevant changes in any of the variables. Additionally, the System Usability Scale (SUS) (0–100) of the VR system was rated positively (SUS = 75.3 ± 4.5), supporting its applicability in educational contexts.

**Conclusions:**

Both virtual reality training and theoretical instruction were effective in improving cardiopulmonary resuscitation knowledge, attitudes, and self-efficacy among adolescents. However, virtual reality showed a greater long-term retention of effects and received high usability ratings, reinforcing its potential as an educational tool in school settings.

## Introduction

Cardiopulmonary resuscitation (CPR) training is an essential pillar in the global strategy for improving survival after a cardiac arrest. Many international initiatives, such as the *Kids Save Lives* program, backed by the *International Liaison Committee on Resuscitation* (ILCOR), promotes the systematic teaching of basic resuscitation maneuvers at schools from early ages, in order to create a population that is better prepared and able to act in emergency situations.^1^

Traditionally, CPR training has been taught through theoretical and demonstrative sessions, with a variable efficacy in terms of long-term retention, attitude towards helping, and self-confidence to act. In the last few decades, the incorporation of digital technologies has opened new possibilities in health education. Virtual reality (VR) has been shown to be a promising tool to create emotionally-engaging and technically-controlled immersive learning experiences.^2^

The use of virtual environments has been associated to improvements in clinical performance, adherence to guides, and retention of contents, especially in youth and students without previous experience.^3^ According to the *European Resuscitation Council* (ERC) guides, educational methodologies must combine pedagogic effectiveness, accessibility, and sustainability, to achieve a real impact on the acquisition of resuscitation skills.^4^ The accumulated evidence supports the idea that VR can favor these objectives, as it increases motivation, facilitates situated learning, and allows for deliberate repetitions in safe environments.^5^, ^6^, ^7^

However, despite its potential, the superiority of VR as compared to traditional methods in adolescent populations is still being debated.^8^, ^9^ Some studies have shown positive effects on knowledge and attitude, while others did not find sustained differences in the long term.^10^, ^11^ Due to the above, it is necessary to design rigorous studies, with a longitudinal follow-up that not only assesses the knowledge acquired, but also key psychological variables such as attitude and self-efficacy, associated with the intention to act in real emergencies.^12^ Adolescence is a key stage for the internalization of civil and health skills, and effective educational interventions during this phase can create a lasting impact on the preparation of the community when facing emergencies.^13^

The aim of the present study was to assess the efficacy of an educational intervention in cardiopulmonary resuscitation (CPR) through immersive virtual reality, as compared to conventional theoretical training and a control group without an intervention, on knowledge, attitude, and self-efficacy among secondary school students, with a one-year follow-up. The hypothesis is that the virtual reality training will produce more improvements that are sustained in the three dimensions assessed, as compared to the other two groups.

## Materials and methods

### Study design

A randomized trial was designed with three parallel lines and longitudinal follow-up at four different time points: pre-intervention (T-0), immediate assessment after the intervention (T-Immediate), follow-up after a month (T-Month), and after twelve months (T-Year). The study followed the guidelines from the CONSORT 2025 declaration for parallel randomized trials.^14^

### Participants and sample

The study population was composed of 3rd-year Compulsory Secondary Education (ESO) students, aged between 14 and 15 years old from the Region of Murcia, Spain. The inclusion criteria were: being enrolled in the corresponding year, having the informed consent from their parents or legal tutors, and regularly attending school. The students who declined to voluntarily participate or who had visual or neurological disorders that could make it difficult to safely use the virtual reality devices were excluded. The random assignment was performed individually, in a 1:1:1 ratio, through a sequence of random numbers generated by a computer (RAND function in Excel). The sequence was applied by an independent researcher, unrelated to the intervention and the collection of data, to maintain the allocation concealed. No blinding was applied due to the evident character of the interventions.

**Fig. 1:**
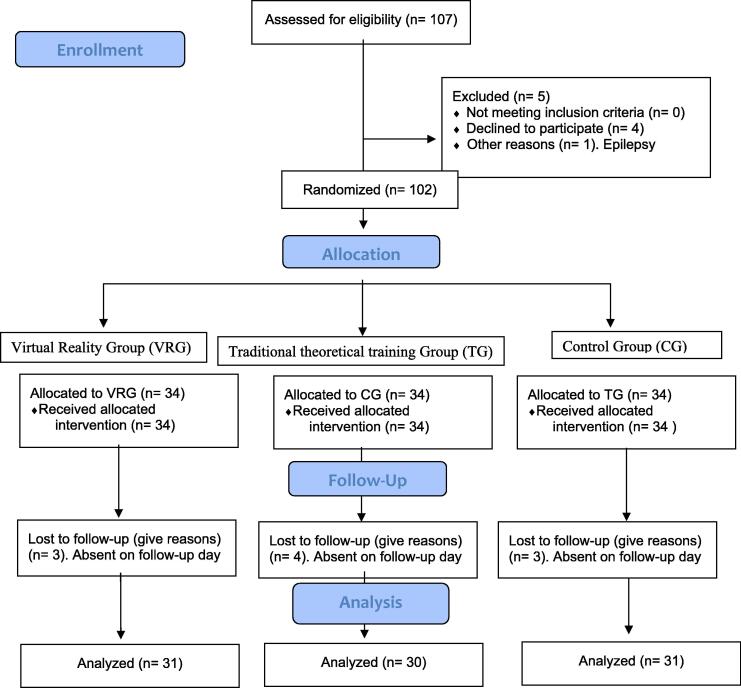
**CONSORT**^14^**flow diagram, with a three-group assignment: Virtual Reality Group (VRG), Traditional theoretical Training Group (TG), and Control Group (CG)**.

The sample size was estimated to detect a moderate-high effect size (*f* = 0.30), with a statistical power of 80 %, and a level of significance of 5 %. A total of 89 students was deemed necessary, and the sample was expanded to 102 students (*n* = 34 per group) to include all the eligible students and to anticipate a drop-out rate of 12 %. After the follow-up, the data from 92 participants were analyzed (Fig. 1): Virtual Reality Group (VRG) (*n* = 31), Traditional theoretical Training Group (TG) (*n* = 30) and Control group (CG) (*n* = 31).

### Interventions

The study groups and the interventions performed were the following:

• **Group 1: Virtual Reality Group.** The participants took part in an immersive experience with Quest 3® goggles and an interactive 360° video in the Wonda VR® platform. A 2D version of the video used in the 3D VR intervention can be watched in the following link: https://www.youtube.com/watch?v=GLZ1VgtMXKQ. The experience simulated a Basic Life Support (BLS) scenario where an adult suffered a cardiac arrest on school grounds. The participant assumed the role of the principal lay rescuer (a student), who, although supported by classmates and teachers, was the sole decision-maker throughout the scenario. The scenario was structured with specific decision points closely aligned with the key steps of the chain of survival. The feedback mechanism was organized as follows: Immediate feedback and correction were provided for critical procedural errors (e.g., failing to verify scene safety or attempting CPR without activating emergency services). This correction was delivered via a 3D superimposed hologram within the scenario, which clearly explained the correct action and its rationale to the user. Summative feedback was given at the end of the scenario, summarizing overall performance and displaying the score obtained from the integrated knowledge questions. The scenario lasted approximately 10 min and covered key learning objectives, including safety assessment, consciousness and breathing checks, activation of Emergency Medical Services (EMS), chest compressions, and correct AED application (Supplementary Material 1).
• **Group 2: Traditional theoretical training Group.** The students received a 10-minute in-person theoretical class about how to act during emergencies, with content that was equivalent to the VR group. This training was performed with the same model as the article by Cristina Cerezo et al. for a theoretical class,^15^ and included the following contents: (1) Chain of survival; (2) Approach to choking, (3) Basic Life Support (BLS) algorithm, and (4) use of the automated external defibrillator (AED).
• **Group 3: Control Group**. This group did not receive training during the study but was assessed at the same time points.

### Variables and instruments

The sociodemographic variables were analyzed through an initial self-administered questionnaire. The variables included were: age, sex, previous experience with CPR training or first aid, previous use of virtual reality goggles, level of familiarity with videogames, self-evaluation of digital skills (scale from 1 to 5). In the other groups, the questionnaires were given digitally at T-0, as well as just after the intervention (T-Immediate), and the follow-ups (T-Month and T-Year).

• **Knowledge**. This is the main study variable. The same ten multiple-choice questions were used in all groups, with identical wording and scoring.
• The only difference was the delivery format: in the VR group, questions were integrated within the Wonda VR® scenario (Supplementary Material 1), whereas in the theoretical and control groups they were answered as a digital questionnaire.
• Both formats used the same 0–10 scoring scale, ensuring direct comparability.
• Although no separate validation study was conducted, all items were reviewed by experts in CPR education to confirm content equivalence. The scenario simulated a situation of basic life support with the use of an AED, including key steps such as safety, consciousness assessment, and breathing, calling 112, chest compressions, and application of the AED. The content and the scoring system were validated by a panel of experts in emergencies and instructional design.^16^
• **Usability.** This variable was only measured in the Virtual Reality group right after the first intervention. It was performed through the use of the *System Usability Scale* (SUS), a validated tool that is widely used in the field of educational and health technology.^17^ The scale is composed of 10 items that explore the perceptions of the user on the ease of use, the complexity, the consistency, and the confidence with the system. Each item is answered with a 5-point Likert-type scale (from 1 = “complete disagreement” to 5 = “complete agreement”), and the total score is converted to a 0–100 scale. Examples of the items include statements such as: “I believe that I would like the use this system frequently”, or “I thought it was unnecessarily complex”.
• **Attitude.** This variable was assessed in all the groups at the four different data collection time points. The attitudes towards the resuscitation were assessed with a Likert-type scale of 5 points, composed of 3 items centered on the willingness of students to act during emergencies, the perception on CPR training, and its usefulness in a school environment.^18^ The items were: “I believe it is important to teach CPR at schools”, “I would like my teachers to teach me CPR”, and “I would be willing to help someone who needs CPR”.
• **Self-efficacy.** The self-efficacy perceived in resuscitation was assessed through a 6-point Likert scale (from 1 = not sure at all to 6 = completely sure), based on an instrument validated by Navalpotro et al., specifically designed for life support training.^19^ For this study, six items centered on key BLS maneuvers and the use of the automated defibrillator (AED) were selected. The participants assessed their level of confidence to perform different actions, such as verifying consciousness and breathing, performing adequate compressions and breaths, and the correct application of an electrical discharge with the AED.

### Statistical analysis

An initial descriptive analysis was performed though means, standard deviations, and 95 % confidence intervals. The normality of the variables was assessed through the Shapiro-Wilk test. For the categorical variables, the Chi-square (Chi^2^) test was used to compare the distribution between the three groups. To analyze the changes within each group over time (T0, T-Immediate, T-Month, and T-Year), a repeated measures ANOVA (RM-ANOVA) was used. To compare the three groups at each time point, a one-way ANOVA was used, followed by a post-hoc Tukey’s test. The effect sizes were expressed as an F value, associated p and partial *η*^2^. The usability scores (SUS) was analyzed with a descriptive statistic. A level of statistical significance of *p* < 0.05 was established. All the analyses were performed with the SPSS v.28 software (IBM Corp.).

### Ethical considerations

All the parents or legal tutors, as well as the school management teams, signed the informed consent. The study was approved by the Research Ethics Committee of the Catholic University of Murcia (UCAM), with code CE112309. Generative Artificial Intelligence tools (OpenAI Data Analyst®) were used exclusively as technical support under the authors’ supervision. Their functions were limited to data organization, verification of descriptive statistics, and assistance in language editing and formatting.

The interpretation of results, statistical analyses, and all scientific conclusions were entirely performed and verified by the research team. No personal or sensitive data were introduced into the AI systems, ensuring compliance with ethical and confidentiality standards. The collection of data was performed between February 6th, 2024, and February 14th, 2025. The T0 and T-Immediate assessments were performed during the Week of Science and Technology of the Floridablanca High School from February 6th to the 9th in 2024, T-Month between March 5th and 9th, 2024, and T-Year during the same Week of Science and Technology of the following year (February 11th to the 14th, 2025).

## Results

### Sample characteristics

Table 1 shows the sociodemographic characteristics of the participating students. No significant differences were observed between the groups in neither the sociodemographic variables nor the previous experience with CPR or technologies.

**Table 1:** Sociodemographic characteristics of the sample.

**Variable**	**Categories** ***n* (%)/Mean ± SD**	**Virtual Reality** **(*n* = 31)**	**Traditional theoretical training group** **(*n* = 30)**	**Control** **(*n* = 31)**	**Test**	***p* value**
**Age**	Mean	14.5 ± 0.6	14.7 ± 0.7	14.8 ± 0.7	ANOVA	0.609

**Sex**	Male	13 (41.9 %)	12 (40.0 %)	12 (38.7 %)	Chi^2^	0.961
	Female	18 (58.1 %)	18 (60.0 %)	19 (61.3 %)		

**Previous CPR experience**	Yes	12 (38.7 %)	13 (43.3 %)	14 (45.2 %)	Chi^2^	0.888
	No	19 (61.3 %)	17 (56.7 %)	17 (54.8 %)		

**Previous use of VR**	Yes	17 (54.8 %)	16 (53.3 %)	16 (51.6 %)	Chi^2^	0.961
	No	14 (45.2 %)	14 (46.7 %)	15 (48.4 %)		

**Frequency of videogame use**	Never	8 (25.8 %)	6 (20.0 %)	7 (22.6 %)	Chi^2^	0.899
	Occasional	9 (29.0 %)	8 (26.7 %)	7 (22.6 %)		
	Frequent	14 (45.2 %)	16 (53.3 %)	17 (54.8 %)		

**Perceived digital skills**	Scale 1–5	3.7 ± 0.7	3.7 ± 0.8	3.7 ± 0.8	ANOVA	0.983

SD: Standard Deviation.

### Changes over time

**Fig. 2:**
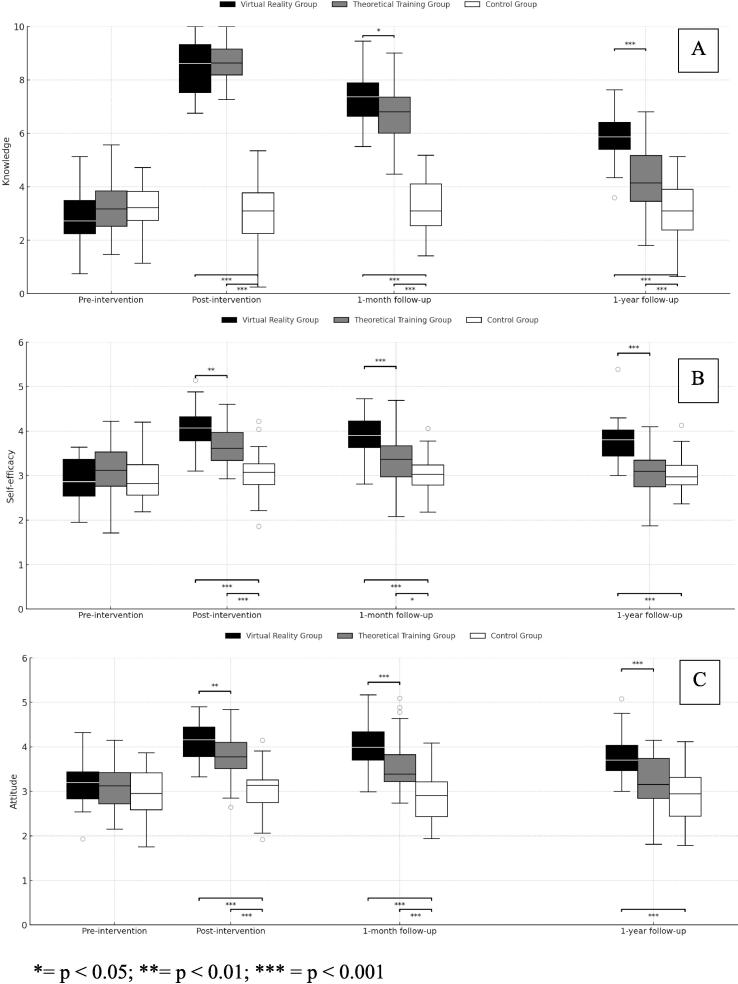
Distribution of results by group and assessment time point. * = *p* < 0.05; ** = *p* < 0.01; *** = *p* < 0.001.

In the intra-group analysis of the variable knowledge (Fig. 2A), significant differences were observed over time in the groups that received the intervention. In the Virtual Reality group, the repeated measures analysis showed a significant variation in the knowledge scores between the different assessment time points (F(3, 90) = 223.56, *p* < 0.001). Similarly, the traditional theoretical training Group also showed significant differences in the change in knowledge throughout the study (F(3, 87) = 184.82, *p* < 0.001). In contrast, the Control group did not show statistically significant changes in their levels of knowledge between the different assessment points (F(3, 90) = 0.50, *p* = 0.685).

With respect to the variable attitude (Fig. 2B), the repeated measures analysis showed significant differences over time in the groups that received the intervention. In the Virtual Reality group, a significant variation was observed between the different assessment times (F(3, 90) = 29.86, *p* < 0.001). The traditional theoretical training Group also showed significant changes in the attitude scores throughout the study (F(3, 87) = 10.20, *p* < 0.001). In turn, the Control group did not show statistically significant differences between the time points assessed (F(3, 90) = 0.60, *p* = 0.615).

No adverse events or incidences related with the intervention were observed in any of the groups. All the analyses were performed according to the protocol, excluding the cases that did not meet the inclusion criteria or had incomplete data during the follow-ups (Fig. 1).

As for the variable self-efficacy (Fig. 2C), the repeated measures analysis revealed significant differences over time in the intervention groups. In the Virtual Reality group, significant variations were identified between the different time points (F(3, 90) = 39.16, *p* < 0.001), and the traditional theoretical training Group also showed significant differences in the levels of self-efficacy throughout the study (F(3, 87) = 7.943, *p* < 0.001). On the contrary, the Control group did not show statistically significant differences between the different assessment times (F(3, 90) = 0.58, *p* = 0.627).

### Comparison between groups at each time point

The differences between groups were observed after the intervention, and were consistent in the three variables analyzed, as shown in Supplementary Material 2. The Virtual Reality group obtained higher scores than the other groups in knowledge, attitude, and self-efficacy, immediately after the intervention, as well as in the follow-ups after a month and a year. In all the cases, the traditional theoretical training Group showed comparable initial improvements, although with a progressive decline over time, while the Control group did not show relevant changes.

Both intervention groups showed a marked increase in the scores after the intervention. In knowledge, for example, the mean score in the Virtual Reality group increased from 3 to 8.5 at T-Immediate, and was maintained at 5.8 after a year, while the traditional theoretical training Group increased from 3.1 to 8.8, and remained at 4.3 after the same period of time. A similar pattern was observed in attitude and self-efficacy, in which the Virtual Reality group practically conserved the improvements obtained after the intervention, while in the traditional theoretical training Group, a more acute decrease was observed during the long-term follow-up.

In this study, the partial *η*^2^ values observed in the repeated measures analysis were especially high in the Virtual Reality group, reaching 0.882 for the variable knowledge, 0.499 in attitude, and 0.566 for self-efficacy, which indicates a large effect size in all cases. In the traditional theoretical training Group, high values were also found, with a partial *η*^2^ of 0.854 in knowledge, 0.260 in attitude, and 0.215 in self-efficacy, confirming the equally considerable effect of the theoretical intervention. In contrast, the values corresponding to the Control group were very low (between 0.016 and 0.02), which suggests a minimum variation explained by the passage of time in absence of the intervention.

### Perceived usability by the Virtual Reality group

In the Virtual Reality group, the perception of usability was assessed with the SUS (*System Usability Scale*). The mean score obtained was 75.3 ± 4.5, based on the answers from 31 participants. This score was higher than the acceptable threshold of acceptable usability (68 points), and corresponded to a qualitative assessment of “good” to “excellent”, according to the international standards for the interpretation of the SUS.^17^

## Discussion

### Main results and interpretation

The present study shows that both intervention methods used in cardiopulmonary resuscitation (CPR) training were efficient in improving the knowledge, attitudes, and self-efficacy of adolescents. The results between virtual reality training and traditional theoretical classes were comparable immediately after the intervention and at the three-month follow-up, but the VR group showed slightly superior outcomes at one year. The effects observed were partially maintained during the follow-up periods, especially self-efficacy, which suggests that VR could contribute to a more lasting internalization of the knowledge.^18^

The results support the initial hypothesis: the VR training was not only efficient in the short-term but also showed a higher retention of the perception of personal skills (self-efficacy**)**, a key aspect in young populations in which subjective confidence has an influence on the willingness to act during emergencies.^19^ The self-efficacy reported by the Virtual Reality group is strongly related to Self-Determination Theory, where VR promotes intrinsic motivation by satisfying the student's psychological needs for autonomy and competence, derived from their role as the sole decision-maker in the simulated scenario.^20^

The most notable difference was observed in the attitude towards resuscitation, in which both intervention groups significantly exceeded the control group, which reinforces the need to include structured training contents even when advanced technology is not available. With respect to the perception of the usability of the virtual reality environment, the results from the *System Usability Scale* (SUS) showed an overall positive score given by the participants, with a mean score of 75 points obtained, which is higher than the acceptability threshold of 68 points established, and corresponding, according to international standards, to a qualitative qualification of “good” and “excellent”.^17^ This high level of usability and positive experience is vital because recent studies confirm that the achievement of immersion is a critical predictors of engagement and learning outcomes in VR/AR medical training.^20^

### Comparison with the literature

In the specific context of BLS training at schools, the ERC^2^ and *Kids Save Lives*^1^ guidelines recommend starting the training at early ages with innovative, accessible, and student-centered methods. The current evidence supports the growing role of immersive technologies in health education. Recent studies have demonstrated that VR can improve motivation, emotional involvement, and the retention of knowledge, as compared the traditional methods.^22^, ^23^, ^24^

Our findings coincide with those reported by Jorge-Soto et al., who demonstrated that practical interventions with adolescents significantly improved their attitude and self-confidence when faced with cardiac arrest situations.^19^ Likewise, the improvement observed in self-efficacy in our intervention groups coincides with that described by Navalpotro et al., who validated specific scales to measure this construct in health training settings, and underlined its predictive value on future behavior.^21^

It must be underlined that although the theoretical teaching produced improvements in all the variables studied, the immersive format was associated with a more sustained subjective perception over time, which is in line with studies that showed that VR facilitates meaningful learning, the mental repetition of the actions, and the making of decisions in real time.^25^

### Strengths and limitations

The present study has methodological limitations that must be considered. Firstly, it was conducted in a single school, which benefitted the follow-up, but restricted the generalization of the findings to other contexts, levels of education, or regions. In addition, although the assignment to the groups was random and managed by an independent researcher, it was not possible to blind for the participants or the personnel, given the evident nature of the interventions, which could have introduced expectation biases.

The variables assessed (attitude and self-efficacy) were measured through self-reported scales, which reflects perceptions rather than real practical skills. No objective measurements were included in the performance of CPR, and no individual factors were controlled for that could have an influence on the learning, such as motivation or the cognitive style. In addition, 5 participants were excluded from the final analysis, 4 of them due to not providing their consent to participate in the study, and 1 of the participants due to having epilepsy. In addition, no variables on practical performance and/or quality of the chest compressions were included. The occurrence of adverse effects related to the use of virtual reality, such as nausea or motion sickness, was also not documented. This is a crucial aspect that needs to be factored into the design of future interventions.^26^

One of the strengths of the study was its longitudinal design, which allowed observing the changes of the participants throughout an entire year. This perspective is particularly relevant in an adolescent population, as adolescents can experience significant changes in their perception, maturity, and attitude, with regard to topics such as resuscitation. In this sense, the control group showed a generalized stability in the three variables assessed, while the groups that received training showed sustained improvements, which suggests that the differences observed were more attributable to the educational intervention, as opposed to a natural maturational process. After the end of the study, the control group was offered the same CPR training intervention, thus ensuring the educational equity among all the participating groups.

### Implications and future lines

The results from the study are aligned with the recommendation found in the Kids Save Lives^1^ document, which promotes the early teaching of resuscitation in school settings, underlining the motivational value of active methodologies and the use of technologies that are attractive to the adolescent population. Virtual reality, in this sense, represents a useful tool for increasing the commitment of students and to facilitate the retention of the knowledge acquired.

The immersive format offers an effective framework for experiential learning that facilitates situated cognition, a mechanism key to long-term retention. Recent literature confirms that the active, body-involved nature of VR training, where knowledge is encoded multisensorially and contextually, is superior to theoretical formats for enhancing long-term retrieval.^27^ Furthermore, the high sense of virtual presence achieved in the VR environment directly influences the emotional encoding of memory, which significantly improves the stability and accessibility of the acquired skill, especially in high-stress medical simulations.^28^

Likewise, the study contributes to reducing the gap in knowledge identified in the ILCOR 2024 review, which underlined the lack of robust data on the effectiveness of virtual reality in CPR training, especially in terms of long-term retention and combination with other methods.^9^, ^29^ Cardiac arrest research has historically received very limited investment, despite its high disease burden.^30^ The results obtained here, with a one-year follow-up and a sustained improvement in the variables assessed, reinforce the potential of these types of instruments as part of blended learning strategies that integrate immersive technology with guided practice and in-person interaction. In this study, the partial *η*^2^ obtained for the variables knowledge, attitude, and self-efficacy in the intervention groups were higher than 0.20 in most of the cases, which reflects a large effect of the education interventions on the variables analyzed. Future studies should incorporate objective measurements of practical skills on CPR and AED use, explore retention in a longer term, and validate these findings with broader and multi-center samples. It would also be valuable to compare different virtual reality formats and assess their relationship with motivational, emotional, and cognitive variables that could mediate the educational impact.

## Conclusion

This study demonstrates that both immersive virtual reality training, as well as conventional theoretical training, are efficient strategies for improving the knowledge, attitude, and self-efficacy perceived on cardiopulmonary resuscitation in the adolescent population. Both interventions generated significant improvements in the short-term, with marked results especially in the Virtual Reality group.

Throughout the one-year follow-up, the virtual reality training group showed a higher stability in the scores, particularly in the perception of personal competence, which suggests a possible advantage in knowledge retention. In addition, the high usability scores reinforce the acceptance and viability in school contexts.

These findings support the use of immersive technologies as a complement to traditional methodologies, in line with the international recommendations with regard to basic life support education from an early age. Future studies must include objective measurements of performance, explore its integration with practical components, and validate these results with broader samples and diverse educational settings.

## CRediT authorship contribution statement

**Ana Belén Ocampo Cervantes:** Writing – review & editing, Writing – original draft, Validation, Investigation, Funding acquisition, Formal analysis, Conceptualization. **Carmen Amalia López López:** Writing – original draft, Methodology, Investigation, Formal analysis, Data curation, Conceptualization. **Cristina Cerezo Espinosa:** Writing – review & editing, Writing – original draft, Investigation, Formal analysis, Data curation. **Robert Greif:** Writing – review & editing, Writing – original draft, Supervision, Methodology, Investigation, Formal analysis, Conceptualization. **Manuel Pardo Rios:** Writing – review & editing, Writing – original draft, Supervision, Project administration, Methodology, Investigation, Funding acquisition, Conceptualization. **Daniel Guillén Martínez:** Writing – review & editing, Writing – original draft, Software, Project administration, Methodology, Investigation, Funding acquisition, Formal analysis.

## Funding

This work was funded by the 10.13039/100014440Ministry of Science, Innovation and Universities in the research project entitled” *Design of a Metaverse for Higher Education in Health Sciences* (PID2022-138884NA-I00), reference MCIN/AEI/10.13039/501100011033/FEDER, UE.

## Data availability

Data is available upon justified request.

## Declaration of competing interest

Robert Greif is the Director of ERC Guidelines and ILCOR, and chair of the ILCOR working group on Education, Implementation, and Teamwork and he is a member of the editorial board of Resuscitation Plus. Manuel Pardo Ríos is a member ILCOR working group on Education, Implementation, and Teamwork and he also is a memmber of the Prehospital Emergency Research Network (RINVEMER) of SEMES, a member of the Spanish Society of Family and Community Medicine (semFYC), and a member of the European Resuscitation Council (ERC). The remaining authors declare no conflicts of interest relevant to the content of this article.
