# Supplementary material for: Virtual reality versus theoretical training in CPR among adolescents: a randomized trial with a one-year longitudinal follow-up
Source: Resusc Plus. 2025 Nov 25;27:101178. doi: 10.1016/j.resplu.2025.101178 (PMC12756614; doi:10.1016/j.resplu.2025.101178)
Supplement: Supplementary Data 1 [file mmc1.docx]

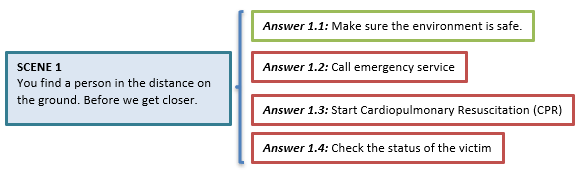

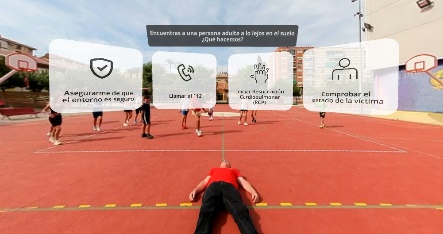


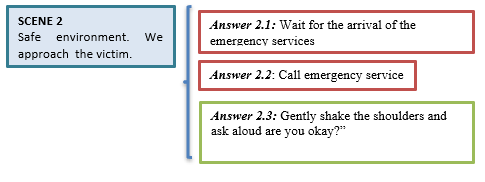

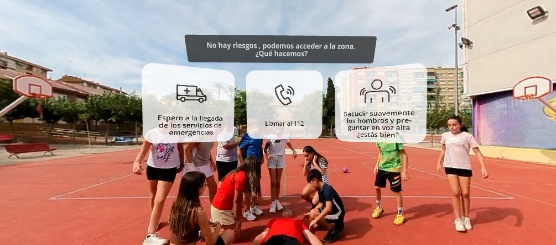


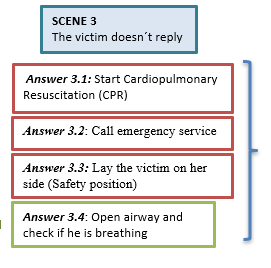

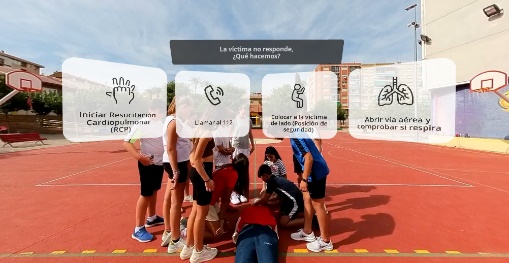


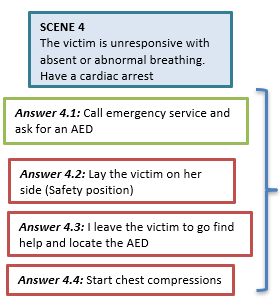

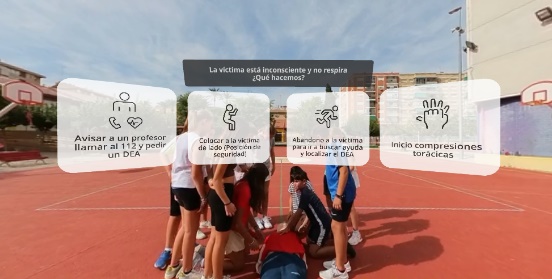


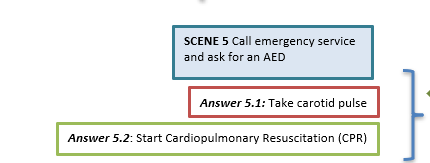

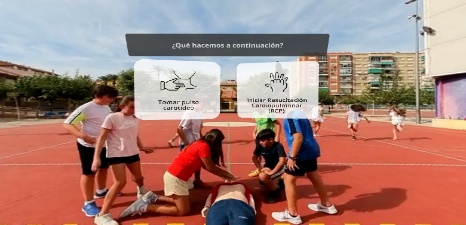


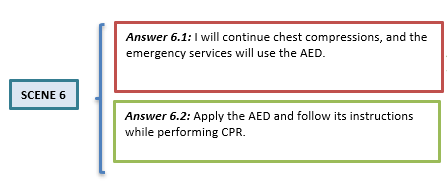

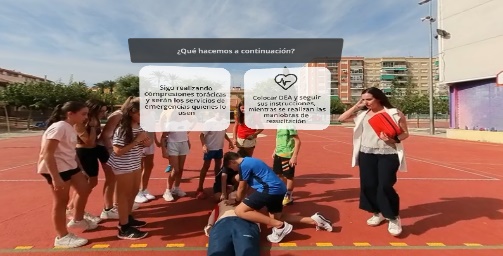


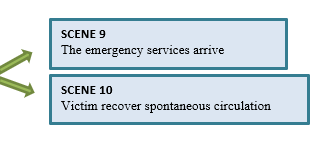


**Supplementary Material 1.** Final version of the decision tree for adult BLS used in both training formats. Left: order of the scenarios; Center: multiple-choice and single-choice questions used in each scene; Right: visual representation on the Wonda VR® platform (screenshot of the virtual environment).

**Supplementary Material 2:** Comparison between groups at each assessment time for knowledge, attitude and self-efficacy.

| **Variable** | **Time point** | **Virtual Reality**  **(n=31)** | **Traditional Theoretical Training Group (n=30)** | **Control**  **(n=31)** | **F** | **p-value** | **Comparisons (Tukey)** |
| --- | --- | --- | --- | --- | --- | --- | --- |
| **Knowledge** | T0-Basal | 3.0±1.0 | 3.1 ± (0.9) | 3.2 ± (0.8) | 0.55 | 0.577 (n.s) | - |
|  | T-Immediate | 8.5 ± (1.0) | 8.8 ± (0.7) | 2.9 ± (1.1) | 352.25 | 5.22 × 10⁻⁴³ (****) | VR > TTT > Control |
|  | T-Month | 7.3 ± (0.9) | 6.6 ± (1.0) | 3.2 ± (0.9) | 150.01 | 3.12 × 10⁻²⁹ (****) | VR > TTT > Control |
|  | T-Year | 5.8 ± (0.7) | 4.3 ± (1.0) | 3.0 ± (1.1) | 62.25 | 1.23 × 10⁻¹⁷ (****) | VR > TTT = Control |
|  | F (gl1, gl2) | F(3, 90) = 223.56 | F(3, 87) = 184.820 | F(3, 90) = 0.497 | - | | |
|  | p-value | 1.39 × 10⁻⁴¹ (****) | 1.26 × 10⁻³⁷ (****) | 0.685 (n.s.) |  |  |  |
|  | Partial η² | 0.882 | 0.854 | 0.016 |  |  |  |
| **Attitude** | T0-Basal | 3.2 ± (0.3) | 3.0 ± (0.5) | 2.9 ± (0.5) | 2.93 | 0.059 | - |
|  | T-Immediate | 4.1 ± (0.4) | 3.7 ± (0.4) | 3.0 ± (0.5) | 38.42 | 9.37 × 10⁻¹³ (****) | VR > TTT > Control |
|  | T-Month | 4.1 ± (0.4) | 3.6 ± (0.5) | 2.9 ± (0.5) | 42.53 | 1.09 × 10⁻¹³ (****) | VR > TTT > Control |
|  | T-Year | 3.7 ± (0.4) | 3.2 ± (0.5) | 2.9 ± (0.5) | 20.69 | 4.18 × 10⁻⁸ (****) | VR > TTT = Control |
|  | F (gl1, gl2) | F(3, 90) = 29.857 | F(3, 87) = 10.201 | F(3, 90) = 0.602 | - | | |
|  | p-value | 1.72×10⁻¹³ (****) | 7.98×10⁻⁶ (****) | 0.615 (n.s.) |  |  |  |
|  | Partial η² | 0.499 | 0.26 | 0.02 |  |  |  |
| **Self-efficacy** | T0-Basal | 2.8 ± (0.5) | 3.1 ± (0.5) | 2.9 ± (0.4) | 1.95 | 0.149 (n.s) | - |
|  | T-Immediate | 4.0 ± (0.4) | 3.7 ± (0.4) | 3.0 ± (0.5) | 38.48 | 9.07 × 10⁻¹³ (****) | VR > TTT > Control |
|  | T-Month | 3.8 ± (0.) | 3.3 ± (0.6) | 3.0 ± (0.4) | 22.21 | 1.50 × 10⁻⁸ (****) | VR > TTT > Control |
|  | T-Year | 3.8 ± (0.4) | 3.1 ± (0.4) | 3.0 ± (0.4) | 30.02 | 1.09 × 10⁻¹⁰ (****) | VR > TTT = Control |
|  | F (gl1, gl2) | F(3, 90) = 39.162 | F(3, 87) = 7.943 | F(3, 90) = 0.584 | - | | |
|  | p-value | 2.75×10⁻¹⁶ (****) | 9.66×10⁻⁵ (****) | 0.627 (n.s.) |  |  |  |
|  | Partial η² | 0.566 | 0.215 | 0.019 |  | | |

n.s.: non-significant; VR = Virtual Reality; TTT: Traditional theoretical training; n.s.: not significant; ****:p < 0.0001
